# Thrombin regulation of synaptic transmission and plasticity: implications for health and disease

**DOI:** 10.3389/fncel.2015.00151

**Published:** 2015-04-21

**Authors:** Marina Ben Shimon, Maximilian Lenz, Benno Ikenberg, Denise Becker, Efrat Shavit Stein, Joab Chapman, David Tanne, Chaim G. Pick, Ilan Blatt, Miri Neufeld, Andreas Vlachos, Nicola Maggio

**Affiliations:** ^1^Department of Neurology, The J. Sagol Neuroscience Center, The Chaim Sheba Medical CenterTel HaShomer, Israel; ^2^Institute of Clinical Neuroanatomy, Neuroscience Center Frankfurt, Goethe-University FrankfurtFrankfurt, Germany; ^3^Department of Neurology, The Sackler School of Medicine, Tel Aviv UniversityTel Aviv, Israel; ^4^Department of Anatomy and Anthropology, The Sackler School of Medicine, Tel Aviv UniversityTel Aviv, Israel; ^5^Department of Neurology and Epilepsy Unit, The Tel Aviv Sourasky Medical CenterTel Aviv, Israel; ^6^Talpiot Medical Leadership Program, The Chaim Sheba Medical CenterTel HaShomer, Israel

**Keywords:** thrombin, proteases activated receptor 1 (PAR1), clotting factors, long term potentiation, synaptic plasticity, hippocampus

## Abstract

Thrombin, a serine protease involved in the blood coagulation cascade has been shown to affect neural function following blood-brain barrier breakdown. However, several lines of evidence exist that thrombin is also expressed in the brain under physiological conditions, suggesting an involvement of thrombin in the regulation of normal brain functions. Here, we review ours’ as well as others’ recent work on the role of thrombin in synaptic transmission and plasticity through direct or indirect activation of Protease-Activated Receptor-1 (PAR1). These studies propose a novel role of thrombin in synaptic plasticity, both in physiology as well as in neurological diseases associated with increased brain thrombin/PAR1 levels.

## Thrombin in the Blood and in the Brain

**Figure 1:**
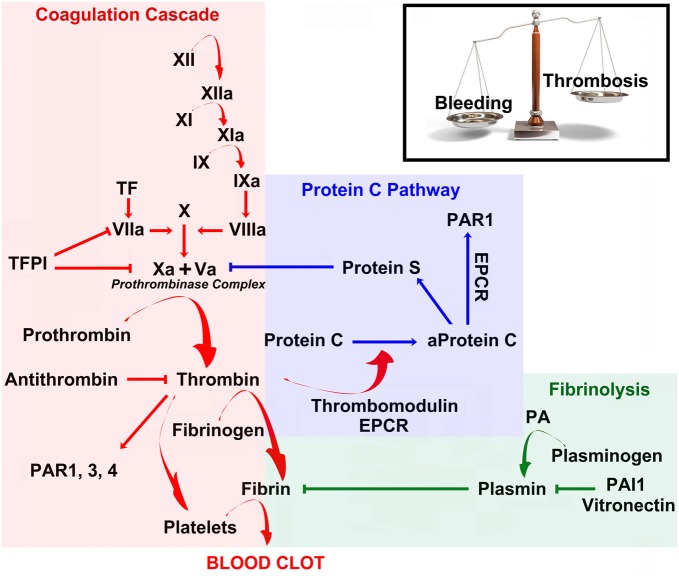
**Coagulation, anticoagulation and fibrinolysis maintain a delicate physiological balance between bleeding and thrombosis**. Schematic diagram of the coagulation cascade (red), Protein C pathway (blue) and fibrinolysis (green). See main text for more detailed explanation. Abbreviations: TFPI, Tissue Factor Pathway Inhibitor; PAR, Proteases activated receptors; EPCR, Endothelial Protein C Receptor; PA, Plasminogen Activator; PAI, Plasminogen Activator Inhibitor.

Coagulation is a fundamental biological process, by which cellular as well as protein components in the blood form a clot to stop bleeding from injured vessels (Kalz et al., 2014). It consists of a cascade of molecular events leading to the activation of thrombin, which catalyzes the conversion of fibrinogen to fibrin, the building blocks of the hemostatic plug (Figure 1; Siller-Matula et al., 2011; Lippi et al., 2012). Thrombin is a serine protease which is activated by the enzymatic cleavage of two sites on prothrombin by activated Factor X (FXa; Furie and Furie, 2005). The activity of FXa is enhanced by binding to activated Factor V (FVa, which forms the prothrombinase complex with FXa; Figure 1; Furie and Furie, 2005).

Prothrombin is produced in the liver and is post-translationally modified in a vitamin K-dependent reaction that converts 10 glutamic acids on prothrombin to gamma-carboxyglutamic acid (Gla; Huang et al., 2003). In the presence of Ca^2+^, the Gla residues promote the binding of prothrombin and other coagulation factors to exposed phospholipid bilayers, which accelerates but also restricts clotting procedure in a dose-dependent manner to injured sites (Huang et al., 2003). Deficiency of vitamin K or administration of the anticoagulant *warfarin*, an inhibitor of the vitamin k epoxide reductase, inhibits the production of Gla residues and slows the activation of coagulation (Huang et al., 2003). As part of its activity in the coagulation cascade, thrombin also promotes platelet-activation and aggregation (Figure 1; Borissoff et al., 2011).

Conversely, thrombin initiates a feedback mechanism which leads to its own inhibition. Once bound to thrombomodulin, an integral membrane protein expressed by endothelial cells, it increases its affinity to and activates Protein C. Activated Protein C (aPC) in turn leads to the inactivation of the prothrombinase complex (= FXa + FVa) at sites with an intact endothelium (Figure 1; Mosnier et al., 2014). In addition, freely circulating thrombin is blocked by antithrombin-III (Figure 1), a serine protease inhibitor, which is enhanced in its activity by the anticoagulant *heparin* (Jo et al., 2014). Taken together, blood thrombin activity is tightly regulated by a set of positive- and negative-feedback mechanisms, which promote clotting at injured sites and prevent coagulation at healthy sites. Considering its key role in the coagulation cascade it is not surprising that novel oral anticoagulants (NOACs) have been developed that act as direct (i.e., dabigatran) or indirect thrombin inhibitors (i.e., rivaroxaban and apixaban, via FXa inhibition) (Levy et al., 2014).

Beyond its role in the dynamic process of blood clot formation, thrombin has pronounced pro-inflammatory effects (Esmon, 2014). Acting via specific cell membrane receptors, the Protease-Activated Receptors (PARs), which are abundantly expressed in all arterial vessel wall constituents, thrombin has the potential to exert pro-atherogenic actions, such as leukocyte migration, cellular proliferation, regulation of vascular permeability and tone, platelet-activation, and edema formation (Coughlin, 2000, 2001; Sambrano et al., 2001; Chen and Dorling, 2009; Schuepbach et al., 2009; Spiel et al., 2011). PARs belong to a unique family of G protein-coupled receptors (Luo et al., 2007). Their activation is initiated by an irreversible, site-specific proteolytic cleavage in the N-terminal extracellular region, which uncovers a tethered ligand activating Gα_q/11_, Gα_i/o_, or Gα_12/13_ -proteins (Coughlin, 2000; Macfarlane et al., 2001; Traynelis and Trejo, 2007). Activation of PARs can recruit multiple intracellular signaling pathways depending on the activating ligand (Russo et al., 2009). This agonist-biased signal transduction and the resulting diversity of intracellular signaling pathways appear to be crucial for the multiple actions of PARs (Russo et al., 2009; Bourgognon et al., 2013).

Interestingly, PARs are also expressed in the brain and while PAR2 represents a class of trypsin/tryptase-activated receptors, PAR1, PAR3, and PAR4 are most effectively activated by thrombin (Gingrich and Traynelis, 2000). In the brain, PAR1 has been detected in both neurons and astrocytes, with the latter demonstrating stronger immunohistochemical signal in human brain tissue (Junge et al., 2004). High levels of PAR1 are detected in the hippocampus, cerebral cortex, and striatum of humans (Junge et al., 2004). While the precise molecular pathways activated by neural PAR1 are yet under investigation, in the brain PAR1-activation has been shown to modulate synaptic transmission and plasticity through the enhancement of N-methyl-D-aspartate receptor (NMDAR) currents (Gingrich et al., 2000; Lee et al., 2007; Maggio et al., 2008; Becker et al., 2014; Vance et al., 2015). In addition, PAR1-deficient animals have been reported to have alterations in hippocampus-dependent learning and memory processes (Almonte et al., 2007, 2013), indicating that PAR1 plays a critical role in memory formation and synaptic plasticity under physiological conditions.

A variety of neurological conditions have been associated with changes in the expression of PAR1 in the brain. In Parkinson’s disease, a significant increase in the number of astrocytes expressing PAR1 has been reported in the substantia nigra pars compacta (Ishida et al., 2006). In addition, upregulation of PAR1 in astrocytes has been observed in HIV encephalitis (Boven et al., 2003), implicating this receptor in neuroinflammatory responses. This idea is supported by the evidence of elevated levels of thrombin in an experimental model of multiple sclerosis (Beilin et al., 2005) and other inflammatory brain diseases (Chapman, 2006). Stimulation of PAR1 by thrombin causes proliferation of glia and produces reactive gliosis, infiltration of inflammatory cells, and angiogenesis (Striggow et al., 2001). Finally, expression of PAR1 is increased in experimental models of Alzheimer’s disease (Pompili et al., 2004) and brain ischemia (Striggow et al., 2001).

**Figure 2:**
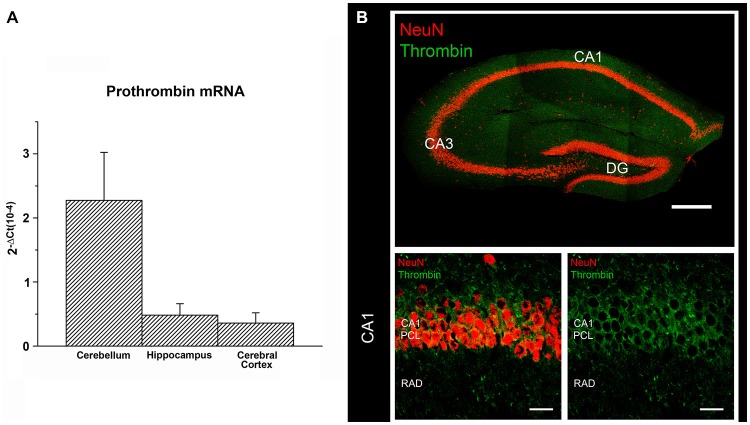
**Prothrombin and Thrombin are expressed in the brain. (A)** Prothrombin mRNA measured by (RT)qPCR in cerebellum, hippocampus and cerebral cortex of C57BL/6J mice (primers: 5′ CCGAAAGGGCAACCTAGAGC, 5′ GGCCCAGAACACGTCTGTG). The results were normalized to HPRT gene expression within the same cDNA sample and calculated using the ΔC_T_ method with values being expressed as 2^−ΔCt^. **(B)** Immunostaining for thrombin (Santa Cruz Biotech, goat polyclonal IgG, cleaved thrombin HC (m361), sc-23335; dilution 1:100 in PBS-based solution) and NeuN (Millipore, mouse monoclonal, MAB377, dilution 1:1000 in PBS-based solution) in the dorsal hippocampus of adult C57BL/6J-mice. Thrombin labeling is predominantly detected in the pyramidal cell layer (PCL) of CA1. The fiber-layers (e.g., stratum radiatum, rad) display weaker but more clustered immunohistochemical signal for thrombin. Scale bar at low magnification = 300 μm; scale bar at higher magnification = 20 μm.

Both thrombin and its inactive precursor prothrombin have been also detected in the brain (Dihanich et al., 1991; Xi et al., 2003). Prothrombin mRNA shows the highest expression in the cerebral cortex and a moderate expression in the hippocampus and cerebellum (Figure 2A; Dihanich et al., 1991). In the hippocampus pronounced immunohistochemical labeling of thrombin is observed in pyramidal cell layers, while lower (clustered) thrombin signal is seen in the fiber-layers (Figure 2B). Although the precise cellular source of thrombin in the brain and the molecular mechanisms responsible for its formation and release warrant further investigation, experimental evidence has been provided that neural prothrombin expression and thrombin activity are highly regulated under physiological and pathological conditions (Xi et al., 2003; Stein et al., 2015). Hence, the molecular machinery of thrombin/PAR1 signaling is detected not only in the vascular system but also present in brain tissue, where it seems to act as a modulator of neural plasticity.

## Concentration-Dependent Effects of Thrombin on Synaptic Plasticity

Long term potentiation (LTP) or depression (LTD) of synaptic strength, homeostatic plasticity and metaplasticity are considered to play important roles for the ability of the brain to effectively learn and adapt to novel challenges (Malenka, 2003; Turrigiano, 2012; Hulme et al., 2013). Consistent with the crucial role of synaptic plasticity in brain function (and similar to the blood coagulation cascade), plasticity is a highly regulated process composed of multiple feed-forward and feed-back mechanisms. This situation tunes neural networks in a way that promotes stability, but at the same time allows for rapid and site-specific responses; including changes in the threshold, direction and duration of synaptic events. Conceptually this *stable dynamic state* of synaptic plasticity is comparable to the blood coagulation cascade, which is kept in a delicate balance between bleeding and site-specific thrombosis. Therefore, it has been hypothesized that similar signaling pathways are employed in blood coagulation and synaptic plasticity.

Indeed, robust experimental evidence exists that thrombin and other coagulation factors are involved in the regulation of LTP in the hippocampus (Gingrich et al., 2000; Almonte et al., 2007; Maggio et al., 2008, 2013b,c; Mannaioni et al., 2008; Hamill et al., 2009). Furthermore, it has been shown that PAR1-signaling mediates thrombin-induced synaptic plasticity, which requires the activation of NMDARs (Lee et al., 2007; Han et al., 2011; Oh et al., 2012; Park et al., 2013).

**Figure 3:**
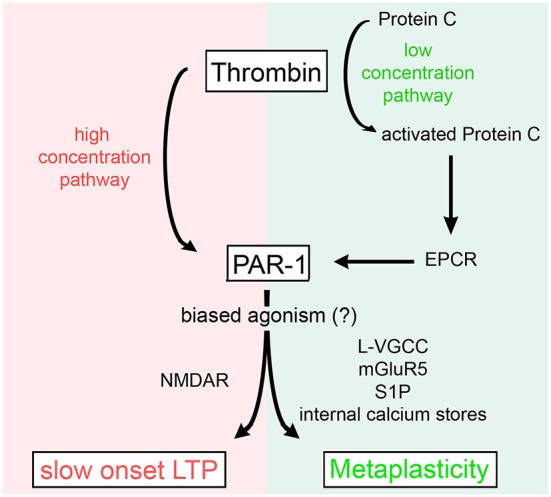
**Concentration-dependent effects of thrombin on synaptic plasticity**. High levels of thrombin cause a slow onset NMDAR-dependent LTP by direct activation of PAR1. Low concentrations of thrombin activate Protein C which binds to EPCR and activates PAR1 to induce metaplasticity, i.e., a reduction in the LTP threshold [by recruitment of L-type voltage gated calcium channels (L-VGCC), metabotropic glutamate receptors 5 (mGluR5), sphingosine-1-phosphate (S1P), and internal calcium stores]. For further details refer to the main text and Maggio et al. (2013c).

In a recent attempt to shed more light on the physiological vs. pathological functions of thrombin and PAR1 in the brain, we studied the effects of different concentrations of thrombin and PAR1 activating peptide (PAR1-AP) on hippocampal LTP. This set of experiments disclosed that thrombin regulates the threshold for synaptic plasticity in a concentration-dependent manner (Figure 3; Maggio et al., 2013c). Specifically, [Thrombin]_high_ induced an NMDAR-dependent slow onset LTP, which occluded the ability of neurons to express further LTP (Maggio et al., 2008, 2013c). Conversely, [Thrombin]_low_ promoted L-type voltage gated calcium channel (L-VGCC), metabotropic glutamate receptor 5 (mGluR-5), and intracellular Ca^2+^ store-dependent LTP, which required aPC and the activation of the Endothelial Protein C Receptor (EPCR, detected on astrocytes and neurons) (Maggio et al., 2013c). In a follow-up study, we further clarified the role of aPC and EPCR in regulating [Thrombin]_low_-mediated synaptic plasticity (Maggio et al., 2014). We demonstrated that aPC induces metaplasticity and thereby enhances the ability to induce LTP (Maggio et al., 2014). This effect of aPC is mediated by EPCR-dependent PAR1-activation that triggers the production of Sphingosine-1-Phosphate (S1P). In turn, the activation of S1P-receptor 1 and intracellular Ca^2+^ stores, ultimately lead to a reduction in the LTP threshold (Maggio et al., 2014). Hence, high concentrations of thrombin saturate the ability of neurons to express further LTP via direct PAR1-activation (Maggio et al., 2008), while at low concentration thrombin mediates metaplasticity by enhancing LTP via aPC-EPCR mediated PAR1-signaling (Figure 3; Maggio et al., 2013c).

This finding is of considerable interest in clinical settings of cerebrovascular events: upon blood-brain barrier (BBB) opening, exposure to blood-derived thrombin and aPC could interfere with endogenous, i.e., neural thrombin/aPC mediated synaptic plasticity. On the one hand high thrombin concentrations may affect the ability of the brain to use LTP-like plastic processes for acquisition of new “memories”, due to the [Thrombin]_high_-mediated saturation of LTP. On the other hand aPC/PAR1 mediated synaptic plasticity (Maggio et al., 2013c, 2014), could facilitate LTP through other mechanisms and may counteract the [Thrombin]_high_ effect (Maggio et al., 2008). The precise role and interplay of thrombin vs. aPC mediated synaptic plasticity under physiological and pathological conditions and the biological consequences for the course of diseases is currently under investigation.

## PAR1-Activation Affects Homeostatic Synaptic Plasticity

To learn more about the role of thrombin/PAR1-signaling under pathological conditions and to study its role in other forms of synaptic plasticity, we used the entorhinal denervation *in vitro* model (Del Turco and Deller, 2007) and assessed the role of PAR1 in *denervation-induced homeostatic synaptic plasticity* (Vlachos et al., 2012). This model takes advantage of the highly organized entorhino-hippocampal projection, which originates in the entorhinal cortex, forms the perforant pathway, and terminates in the hippocampus. Lesioning this fiber tract results in the partial denervation of neurons in the hippocampus, without directly damaging the dendritic tree of target neurons (Müller et al., 2010). In turn, neurons respond to the loss of entorhinal input with a slow adaptive, i.e., compensatory increase in excitatory synaptic strength (Vlachos et al., 2012, 2013a,b), which is considered to play an important role in stabilizing the activity of neuronal networks under physiological and pathological conditions (Marder and Goaillard, 2006; Maffei and Fontanini, 2009; Turrigiano, 2012; Vitureira et al., 2012). While the presence of PAR1 was not required for homeostatic plasticity of excitatory synapses to occur, our study revealed that PAR1-activation occludes the ability of partially denervated neurons to increase their excitatory synaptic strength in a compensatory manner (Becker et al., 2014). Thus our results provided first experimental evidence that PAR1 activity affects neural homeostasis, which could be of relevance for neurological diseases associated with increased brain thrombin concentrations, neuronal cell loss and denervation of connected brain regions (see also Maggio and Vlachos, 2014).

## The Role of Thrombin/PAR1-Signaling in Ischemic LTP

Finally, in a recent study we demonstrated that thrombin/PAR1-signaling has a fundamental role in oxygen glucose deprivation (OGD)-mediated alterations in synaptic plasticity (Stein et al., 2015). A brief (i.e., a few minutes) deprivation of oxygen and glucose, as seen for example in the early stage of acute ischemia or transient ischemic attack, potentiates synaptic transmission at hippocampal CA3-CA1 synapses in an NMDAR-dependent manner and induces ischemic LTP (iLTP) without causing neuronal cell death (Crépel et al., 1993; Hsu and Huang, 1997; Calabresi et al., 2003). It has been proposed that iLTP could have a major impact on the functional reorganization of neuronal networks during the early phase of ischemic stroke (Calabresi et al., 2003). In this context, we were able to demonstrate that OGD increases thrombin activity in neural tissue, i.e., in acute hippocampal slices, and triggers the induction of iLTP. Moreover, we showed that the induction of iLTP impairs the ability of neurons to express further LTP in a thrombin/PAR1-dependent fashion. Indeed, iLTP was blocked when thrombin activity or PAR1 signaling were inhibited and the ability of neurons to express LTP was rescued (Stein et al., 2015). We concluded from this series of experiments that iLTP resembles a [Thrombin]_high_-mediated LTP and hypothesized that counteracting thrombin signaling in the brain during the acute phase of stroke may improve synaptic plasticity (Stein et al., 2015).

Together, these studies provided robust experimental evidence that PAR1-signaling (which is activated, e.g., by thrombin or aPC) is involved in the regulation of distinct forms of synaptic plasticity under physiological and pathological conditions.

## A Role for Thrombin/PAR1 in Epileptogenesis

Although serine proteases are expressed at very low concentration in neural tissue (Luo et al., 2007), their levels increase significantly in the brain following BBB breakdown. In this condition, a large, non-selective increase in the permeability of brain capillaries and tight junctions takes place, such that high molecular weight proteins (Ballabh et al., 2004) and other blood components gain access to the brain. This particular event occurs in several neurological conditions (Ballabh et al., 2004; Tomkins et al., 2008), including hemorrhagic/ischemic stroke (Hjort et al., 2008; Bang et al., 2009) and traumatic brain injury (Barzó et al., 1997; Tomkins et al., 2008; Itzekson et al., 2014). Even though there is a paucity of information concerning the amount of thrombin crossing the BBB, it has been reported that thrombin levels increase more than 200 fold (from 100 pM to 25 nM) in the cerebrospinal fluid of patients with subarachnoid hemorrhage (Suzuki et al., 1992). In addition, if bleeding occurs directly within the brain tissue, active thrombin and other proteases freely diffuse into the brain parenchyma until blood coagulation closes off the injured vessels (Suzuki et al., 1992). A direct consequence of the contact of thrombin with the brain tissue is the onset of seizures (Lee et al., 1997). In 1997, Lee et al. reported that intracerebral injections of thrombin results in focal motor seizures. Interestingly, the injection of thrombin together with its inhibitor alpha-(2-naphthylsulfonyl-glycyl)-4-amidinophenylalaninepiperidide (alpha-NAPAP) prevented both clinical and electrographic seizures (Lee et al., 1997). Similarly, mice engineered to lack Nexin-1, an endogenous thrombin inhibitor, have an increased susceptibility for kainic acid-induced seizures (Luthi et al., 1997).

Following this line of evidence we were able to demonstrate that thrombin-induced seizures are mediated by activation of PAR1 (Maggio et al., 2008). In hippocampal slices, thrombin at a concentration of 5 nM (1 U/ml) increased spontaneous firing of CA3 pyramidal cells (Maggio et al., 2008). In order to examine whether thrombin facilitates the onset of epileptic discharges in conditions mimicking BBB breakdown in the slice (Chen and Swanson, 2003; Beart and O’Shea, 2007), we exposed neurons to thrombin in presence of elevated [K^+^]_o_ or glutamate. In normal slices, addition of 4 mM [K^+^]_o_ did not produce any noticeable spontaneous seizures, which were clearly seen when [K^+^]_o_ were increased to 15 mM. Similarly, 500 μM but not 100 μM glutamate produced spontaneous seizure-like activity in the slice. Remarkably, thrombin facilitated the response to the lower concentration of [K^+^]_o_ (4 mM) and glutamate (100 μM) to produce seizure-like activity. This facilitatory action of thrombin on the production of seizure-like activity was mediated by PAR1, since it was mimicked by PAR1-AP and blocked by the PAR1-inhibitor SCH79797. However, it did not depend on NMDARs as it was not affected by the selective NMDAR-inhibitors ifenprodil or by 2R-amino-5-phosphonovalericacid (APV), indicating that [Thrombin]_high_-mediated LTP does not explain the acute effects of thrombin on seizure-induction (Maggio et al., 2008). While thrombin induced epileptogenesis seems to be NMDAR-independent, it is possible that thrombin could also indirectly modulate synaptic transmission, e.g., through thrombin-mediated proteolysis and remodeling of the extracellular matrix (e.g., Dityatev, 2010).

More recently, Isaeva et al. (2012) exposed hippocampal slices prepared from young postnatal rats (P6 to P15) to large concentrations of thrombin (10 U/ml), to find that thrombin depolarizes membrane potential of neurons and produces a hyperpolarizing shift of tetrodotoxin-sensitive *INap* through a PAR1-mediated mechanism (Isaeva et al., 2012). In addition, we have reported that thrombin affects synaptic transmission in hippocampal CA3 neurons by enhancing both frequency and amplitude of mEPSCs, while reducing frequency and amplitude of mIPSCs (Maggio et al., 2013b). Together, these studies showed a pro-epileptic effect of thrombin, which induces membrane and synaptic changes leading to seizures via PAR1-activation. However, whether the thrombin-induced increase in neuronal activity leads to the development of epilepsy at a later stage remains unclear. Nevertheless, it has been demonstrated that high concentrations of thrombin induce apoptosis (Xi et al., 2003; Luo et al., 2007), which may lead to maladaptive circuit reorganization and to the development of epilepsy. Additional work is required to test this intriguing possibility and to assess whether thrombin could play a crucial role in epileptogenesis.

## Open Questions and Conclusions

While robust experimental evidence exists that thrombin affects brain functioning through the concentration-dependent regulation of distinct forms of synaptic plasticity, it remains unclear how thrombin exerts such diverse effects by acting on the same receptor, i.e., via PAR1. Conceptually, several possibilities, or a combination of different mechanisms, need to be considered. In endothelial cells for example the recruitment of distinct PAR1-mediated downstream signaling pathways has been reported: while thrombin mediated PAR1-activation recruits G_q_/G_12/13_, aPC seems to act via PAR1-mediated G_i_ (Riewald and Ruf, 2005). This observation is consistent with a study showing that PAR1 interacts with various G proteins (Russo et al., 2009; McCoy et al., 2012). Also, precise location of PAR1 in neuronal or astrocytic membrane compartments and the current state of neuronal networks seem to influence selectivity of PAR1 to distinct ligands and the recruitment of diverse intracellular signaling pathways by PAR1 (Russo et al., 2009; Bourgognon et al., 2013). Such agonist-biased signal transduction may explain the involvement of PAR1 in mediating and influencing various forms of plasticity, depending on the actual thrombin concentration (Figure 3). However, whether distinct subtypes of PAR1 exist, which recruit specific downstream signals, and even show different affinities for thrombin and aPC needs to be tested. The role of simultaneous or selective activation of PAR1 in distinct cell types and/or cellular compartments remains unclear in this context as well. While some evidence for astrocytic PAR1 signaling has been provided (e.g., Vance et al., 2015), the contribution of PAR1 signaling in pre- vs. postsynaptic compartments, in distinct types of interneurons, microglia cells, oligodendrocytes or endothelial cells warrants further investigation. Thrombin may also exert its function through other receptors and proteolysis of specific components in the extracellular matrix (e.g., fibrinogen) under physiological and pathological conditions (e.g., epileptogenesis, see Dityatev, 2010). Indeed, evidence exists that fibrinogen plays an important role in microglia recruitment (Davalos et al., 2012). Hence, it is interesting to speculate that thrombin could modulate neuroinflammatory responses by acting on PAR1 and fibrinogen (Tripathy et al., 2013). Whether thrombin-induced, fibrin-mediated pathways also act on synaptic transmission and plasticity, and the role of PAR1 in this context has not been addressed so far.

Considering the suggested role of thrombin as a major factor for seizures, the cellular source of thrombin in the brain and the molecular machinery responsible for its formation and release needs to be further clarified. Nevertheless, it is interesting to speculate that new oral anticoagulants, which target thrombin could have an unexpected antiepileptic effect (Maggio et al., 2013a), and may restore the ability of neurons to express plasticity under conditions of increased brain thrombin concentrations and PAR1-activity, as seen for example upon BBB breakdown. Its central role in neural plasticity classifies PAR1 as an interesting therapeutic target in thrombin associated pathologies. We are confident that future studies will provide new important insights on the role of thrombin/aPC/PAR1-mediated neural plasticity and excitability and will shed more light on concentration-, ligand-, source-, target-, and state-dependent recruitment of PAR1 signaling cascades. These information may contribute to the development of new strategies to restore brain function under pathological conditions.

## Conflict of Interest Statement

The authors declare that the research was conducted in the absence of any commercial or financial relationships that could be construed as a potential conflict of interest.
